# Quantitative imaging of anti-phase domains by polarity sensitive orientation mapping using electron backscatter diffraction

**DOI:** 10.1038/s41598-017-11187-z

**Published:** 2017-09-07

**Authors:** G. Naresh-Kumar, A. Vilalta-Clemente, H. Jussila, A. Winkelmann, G. Nolze, S. Vespucci, S. Nagarajan, A. J. Wilkinson, C. Trager-Cowan

**Affiliations:** 1 Department of Physics, SUPA, University of Strathclyde, Glasgow, G4 ONG UK; 2 Department of Materials, University of Oxford, Parks Road, Oxford, OX1 3PH UK; 3 Department of Electronics and Nanoengineering, Aalto University, FI-00076 Aalto, Finland; 4 Bruker Nano GmbH, Am Studio 2D, 12489 Berlin, Germany; 5 BAM, Federal Institute for Materials Research and Testing, Unter den Eichen 87, 12205 Berlin, Germany

## Abstract

Advanced structural characterisation techniques which are rapid to use, non-destructive and structurally definitive on the nanoscale are in demand, especially for a detailed understanding of extended-defects and their influence on the properties of materials. We have applied the electron backscatter diffraction (EBSD) technique in a scanning electron microscope to non-destructively characterise and quantify antiphase domains (APDs) in GaP thin films grown on different (001) Si substrates with different offcuts. We were able to image and quantify APDs by relating the asymmetrical intensity distributions observed in the EBSD patterns acquired experimentally and comparing the same with the dynamical electron diffraction simulations. Additionally mean angular error maps were also plotted using automated cross-correlation based approaches to image APDs. Samples grown on substrates with a 4° offcut from the [110] do not show any APDs, whereas samples grown on the exactly oriented substrates contain APDs. The procedures described in our work can be adopted for characterising a wide range of other material systems possessing non-centrosymmetric point groups.

## Introduction

Various material properties such as piezoelectricity, spontaneous polarisation, and plasticity are directly dependent on the crystal structure, and any form of deviation from their perfect crystal lattice could significantly alter their fundamental behaviour^1^. Producing defect free materials is a challenging task especially in the case of heteroepitaxial thin film growth. Irrespective of the substrates, the growth plane, or the growth conditions employed, extended defects such as dislocations, stacking faults and grain boundaries are generally observed in the as–grown layers. In addition to these commonly observed defects; inversion domains (IDs), antiphase domains (APDs) and antiphase boundaries (APBs) have also been identified in several materials; examples include, layered perovskite structured materials^2, 3^ compound semiconductors^4–6^, metallic superlattices^7^ and shape memory alloys^8^. Integrating the functionalities of all of the previously listed materials on a silicon platform is highly sought after to satisfy the demanding requirements for the next few generations of electronic and optoelectronic devices^9^. For example, monolithic integration of A^III^ - B^V^ compounds on Si substrates would provide high-efficient, low-cost multi junction solar cells compatible with CMOS technology. Optimising the performance of such devices will require pioneering growth, and fabrication supported by characterisation techniques for a detailed understanding of defects. Often extended defects are electrically active^10^ and are problematic for minority carrier devices, such as GaAs solar cells, AlGaN-based ultra violet light emitting diodes, transistors and SiC power devices as well as LaSrMnO_3_ based spintronic devices^3^. This is why structural characterisation techniques which are simultaneously rapid to use, non-destructive and structurally definitive on the nanoscale become a prerequisite.

In this article, we demonstrate a novel application of electron backscatter diffraction (EBSD)^11^ in a field emission scanning electron microscope (FE–SEM) to image and quantify APDs in a single crystalline GaP thin film grown on Si substrates. We have chosen GaP as an example to validate the applicability of using EBSD to characterise APDs; nonetheless our non-destructive, and nanoscale technique can be adopted for other material systems, especially those having non-centrosymmetric point groups^2–8^. Please note optimal experimental conditions have to be chosen while using EBSD for samples with low thermal conductivity^12^.

## Antiphase domains in GaP

**Figure 1:**
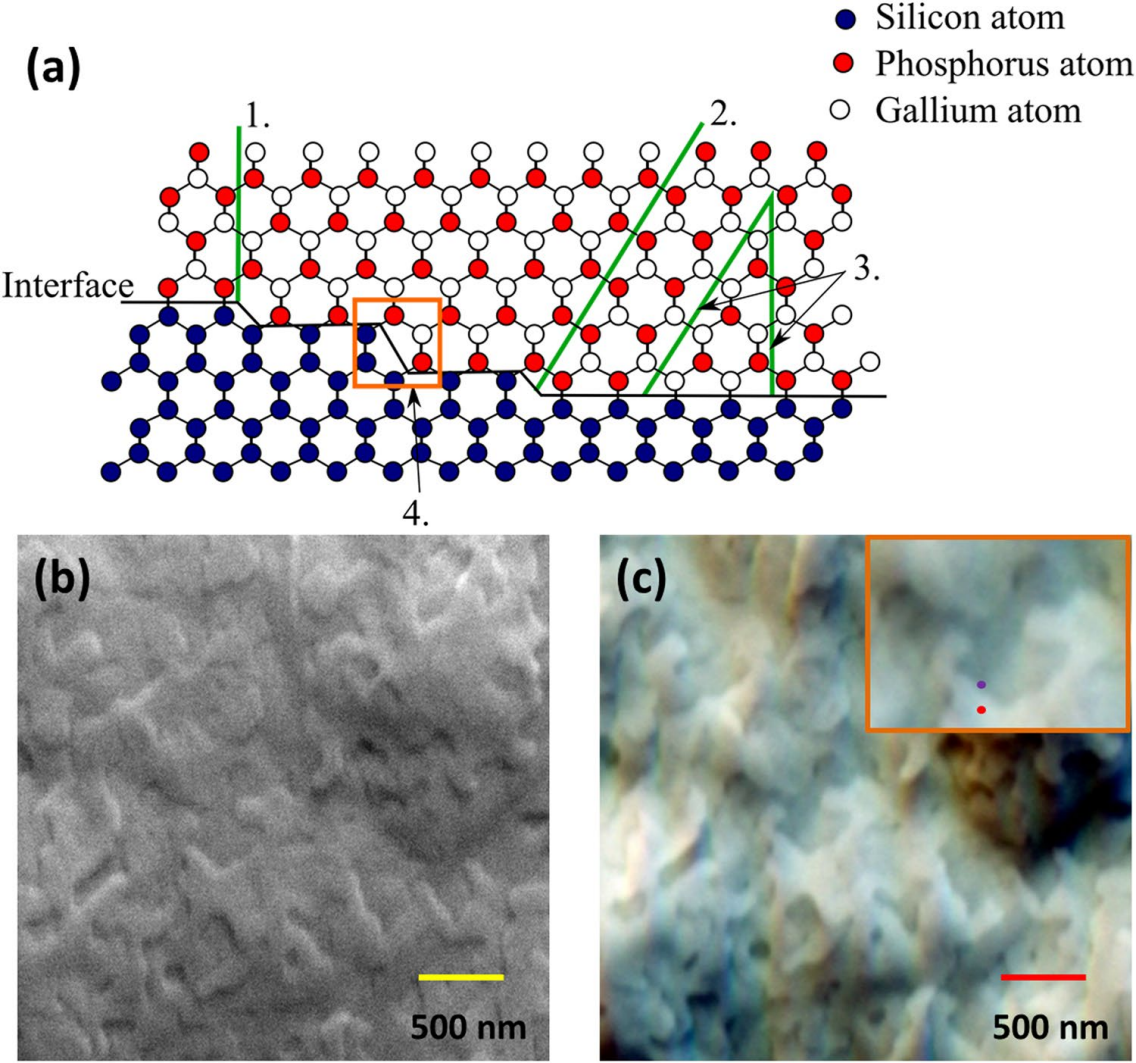
Antiphase domains in GaP on Si substrates. (**a**) Ball and stick model illustrating the formation and annihilation of antiphase boundaries (APBs) in GaP grown on a (001) Si, (1.) APBs parallel to (110) due to sub-lattice occupation disorder, (2.) APBs along the {111}_Si_ due to monoatomic steps, (3.) annihilation of APBs along (111) and (110) and (4.) annihilation of APBs due to diatomic steps. **(b)** Plan-view secondary electron image and (**c**). Plan-view backscattered electron image both acquired at a sample tilt of 70° from the same region of the surface of a 70 nm GaP film grown on an (001) Si substrate (miscut < 0.1°). The inset shows areas marked with a red dot and a purple dot which may well be regions with two orientations corresponding to a difference in the location of cation atoms (eg. Ga) and anion atoms (P) in the two sub-lattices as expected in the case of APDs.

**Figure 2:**
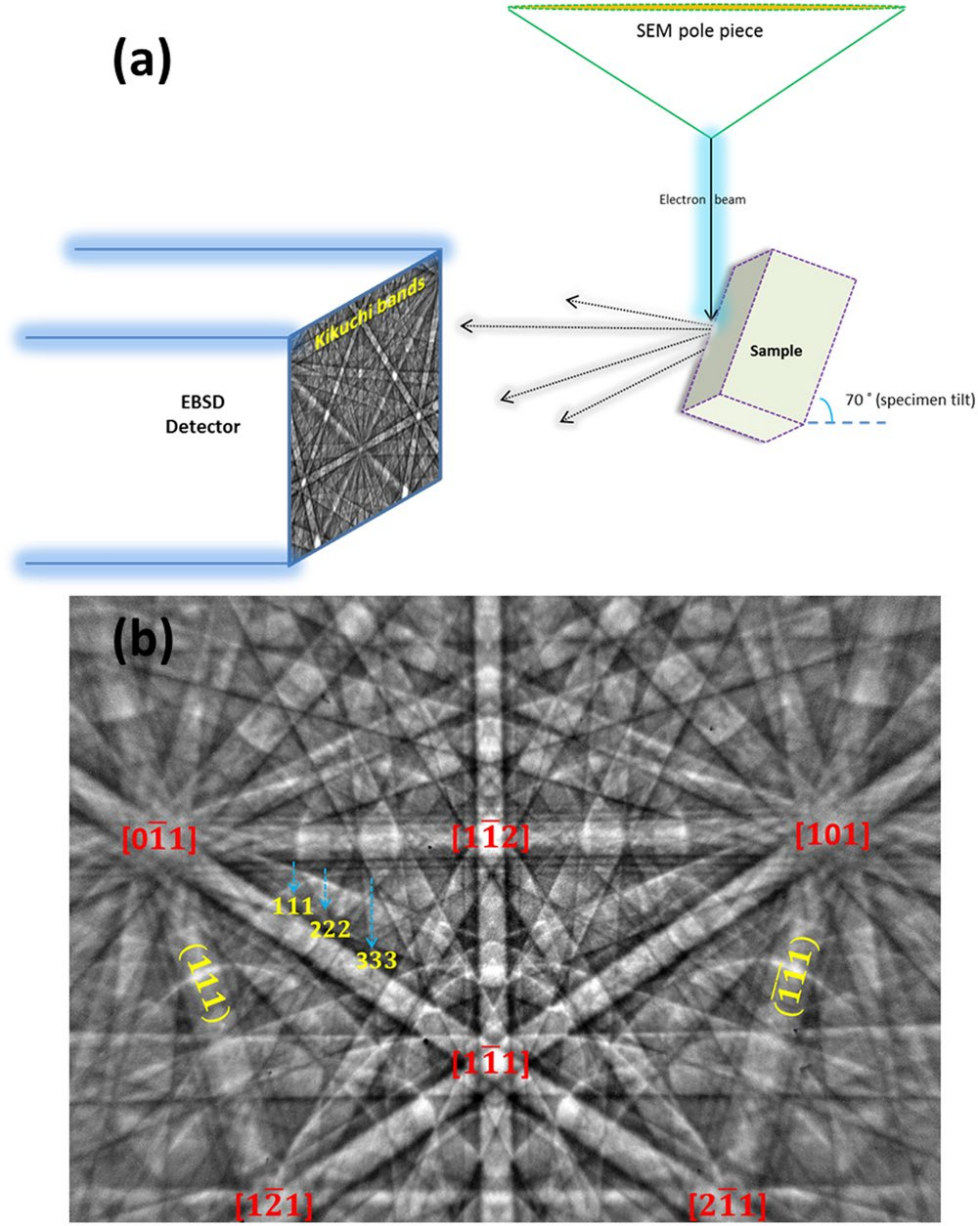
EBSD experimental setup. (**a**) Schematic of a standard EBSD detection geometry and **(b**) EBSD pattern from a GaP thin film marked with some major lattice planes and well-visible zone axes.

**Figure 3:**
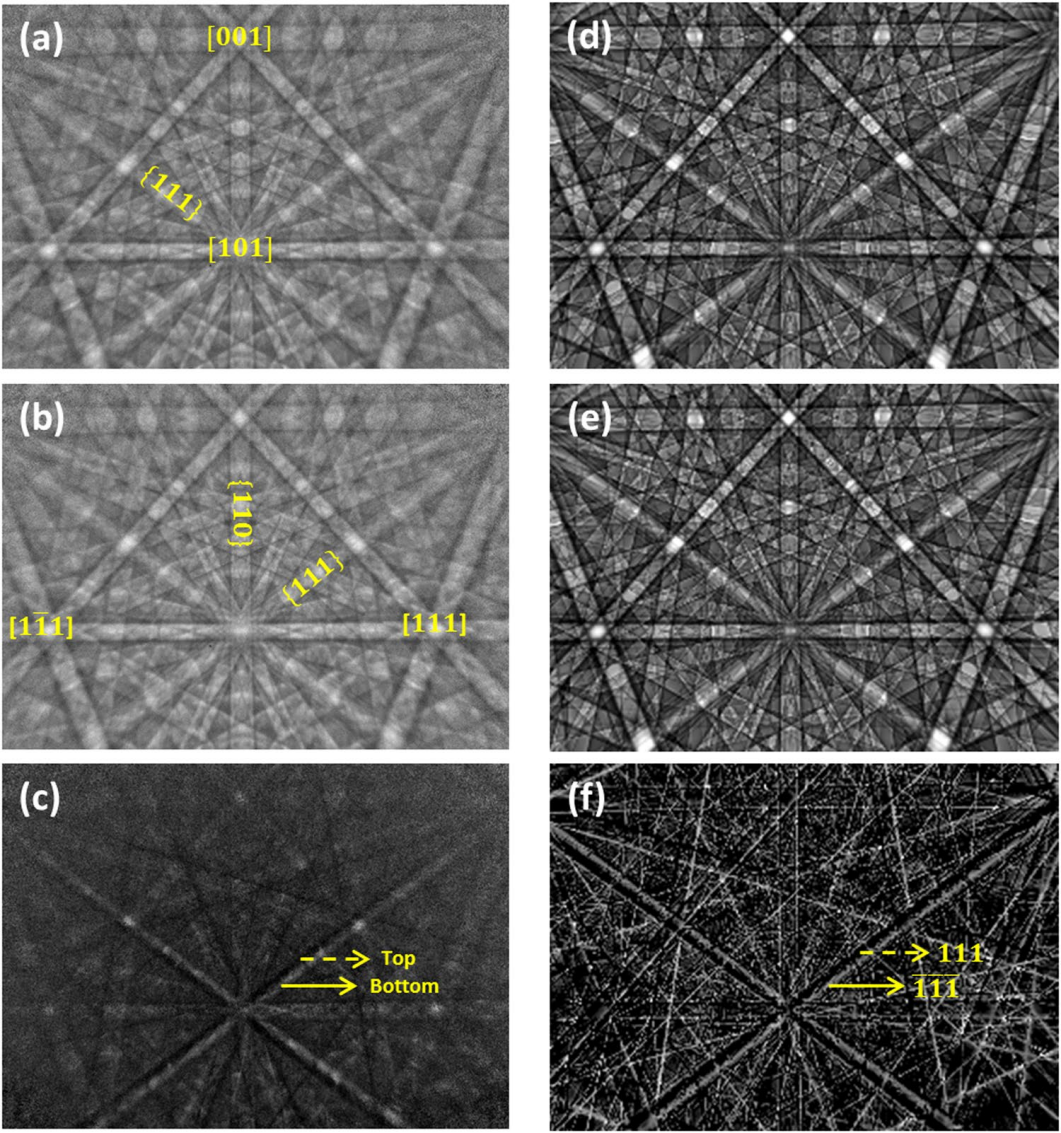
Comparison of experimental and simulated EBSD patterns. (**a**) Experimental EBSD pattern from the red dotted area, see Fig. 1c (for e.g.; the location of cation (Ga) atoms), with r = 0.627 and φ_2_ = 180.9 ° and **(b)** from the purple dotted area (for e.g.: P site with r = 0.627 and φ_2_ = 270.9 °) and **(d** and **e)** the corresponding dynamical simulations. (**c)** Normalised difference intensity image (I_a_−I_b_)/(I_a_ + I_b_) of the two experimental patterns and (**f**) normalised difference intensity image of the two simulated patterns. The strong asymmetric intensity difference between the {111} and $\{\bar{1}\bar{1}\bar{1}\}$ in the normalised difference intensity images clearly indicates the crystal structure rotation by 90° confirming the presence of APDs in the GaP thin film.

**Figure 4:**
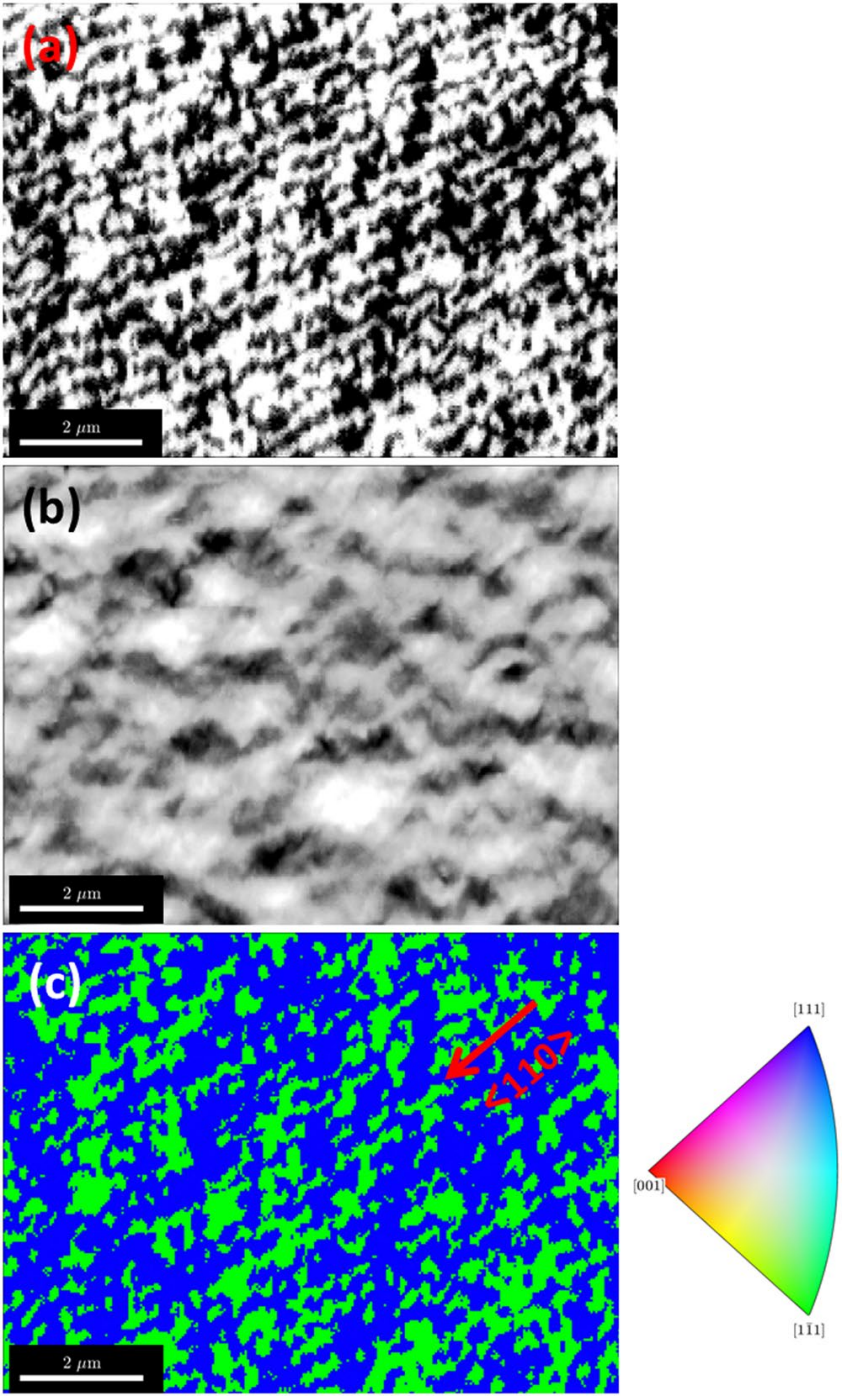
Imaging antiphase domains. **(a**) Region of interest asymmetry imaging from the {111} bands produced from the background corrected EBSD patterns. The bright and dark regions indicate the two different pseudo-symmetric domains, **(b)** corresponding total intensity image of the raw EBSD patterns and (**c**) the EBSD inverse pole figure (IPF) map for the sample reference direction [1,1,1] revealing the APDs. Regions with APDs are coloured green and blue according to the IPF colour key, which indicates the expected 90° misorientation between the two possible domains. The step structures (of the order of 100 nm) can also be seen along $\,\langle 110\rangle$.

When a non-centrosymmetric polar material such as zincblende GaP ($\bar{4}3\,$ *m*) is epitaxially grown on a centrosymmetric non-polar material Si (*m* $\bar{3}\,$ *m*), two equivalent orientations corresponding to a difference in the location of cation atoms (for e.g., Ga) and anion atoms (P) in the two sub-lattices can be formed leading to the creation of APDs. The boundary separating the domains of different sub-lattice location is defined as the APB. Formation of APDs in GaP epilayers grown on Si surfaces is mainly affected by the surface steps of the Si substrate, see Fig. 1a. Atomically clean, vicinal (001) Si surfaces are generally dominated by monoatomic steps due to their low formation energy^13^. The monoatomic steps have a width of ¼ of the Si lattice constant due to the diamond cubic structure (space group Fd$\bar{3}\,$m) with two tetrahedrally connected Si atoms in each primitive cell separated by ¼ of the width of the unit cell in all three dimensions. Typically, these monoatomic steps along the growth direction [001] are assumed to be responsible for the formation of APBs at the Si/GaP interface, leading to their propagation along the (011) plane (see Fig. 1a–1) or the (111) plane (see Fig. 1a–2)^14^. APBs can also be formed due to the sub-lattice occupation disorder at the Si/GaP interface parallel to {110} _Si_ or {111} _Si_, where Ga and P atoms in the region to the left of the boundary sit in different sub-lattices from the region to the right (see Fig. 1a–3). In simple geometric terms, the GaP crystal appears to be rotated by 90° around[001] between the sides of the APBs. APDs could be eliminated either by self-annihilation along the {111}_Si_, by obtaining a uniform monolayer of either Ga or P nucleation on Si by using optimised growth conditions, or by growing on Si surfaces with diatomic steps (see Fig. 1a–4)^15–18^. Nonetheless, it is quite a challenging task to produce smooth GaP surfaces especially below the critical thickness (of order 90 nm for GaP grown on (001) Si^19^), the sample surface can exhibit “twisted-line” like features. The twisted-line morphology can result from the surface step structures from the Si substrate and is associated with APBs^18^. The sample morphology exhibiting the twisted-lines are shown in the secondary electron image (SE) as well as in the backscattered electron image (BSE) acquired in a forward scattered geometry, also referred to as a forescattered electron (FSE) image, see Fig. 1b and c respectively. The images were acquired from a 70 nm thick GaP film grown on an (001) Si substrate (miscut < 0.1°) by metalorganic vapor phase epitaxy. The inset in Fig. 1c is marked with a red dot and a purple dot which may well be regions with two orientations corresponding to a difference in the location of cation atoms (eg. Ga) and anion atoms (P) in the two sub-lattices as expected in the case of APDs.

Although stress induced extended defects such as dislocations are unlikely to form below the critical thickness, non-optimised growth conditions can generate stacking faults. The formation of stacking faults may be due to the coalescence of 3-D islands produced as a result of a lack of charge neutrality along the Si-GaP interface. Possessing equal numbers of Si-Ga bonds and Si-P bonds is essential to maintaining charge neutrality which is needed to initiate 2-D growth, which in turn is a requirement for producing smooth surfaces^17–20^. In the present work we will limit the discussion to imaging APDs in our samples. Nonetheless, we note that the set-up used in this work (i.e. electron diffraction imaging with high resolution SEM) is also suited for observing stacking faults formed due to the coalescence of 3-D islands.

Several methods have previously been reported for detecting APDs in GaP, examples include anisotropic etching, X-ray diffraction (XRD)^21^, reflectance anisotropy spectroscopy (RAS)^22, 23^ and transmission electron microscopy (TEM)^4, 14, 24, 25^. All these methods are either indirect or destructive and time consuming or cannot provide statistically significant information on APDs. In contrast to all the previously reported techniques, the scanning electron microscope (SEM) based electron diffraction techniques of EBSD and electron channelling contrast imaging (ECCI) provide the capability of rapid and non-destructive characterisation, giving accurate wide area crystallographic information with resolution down to the order of tens of nanometres^11, 26–29^.

### EBSD in a scanning electron microscope

In EBSD, an electron beam is incident on a sample which is typically tilted at an angle of 70° towards the detector. Classically the detector is a phosphor screen which captures backscattered electrons from the sample. The quasi-elastically backscattered part produce a diffraction signal, generally referred to as a backscattered Kikuchi diffraction (BKD) pattern or electron backscatter diffraction (EBSD) pattern. The EBSD pattern also holds all the inelastically scattered electrons (background signal). The schematic of the detection geometry and the experimental setup is shown in Fig. 2a. As a first approximation, the visible bands in an EBSD pattern can be interpreted by the angular distribution of the Bragg-reflected electrons coming from the lattice planes (*hkl*) of a crystalline sample. Due to the cylindrical symmetry of the Bragg reflection conditions with respect to the lattice plane normal, diffraction cones are formed. When the diffraction cones (Kossel cones) intersect the phosphor screen, nearly straight lines (Kikuchi lines) are seen due to the shallow angle of the Bragg-diffracted cones. Thus the EBSD pattern can be used to directly measure the crystal’s orientation. An example EBSD pattern acquired from a GaP thin film is shown in Fig. 2b. It is these diffraction patterns which hold the structural information of the crystalline specimen. By moving a focused electron beam point by point across a grid of positions on the sample surface, phase distribution or orientation maps can be derived providing a quantitative representation of the local microstructure.

EBSD patterns represent the gnomonic projection of the diffraction signal. Any rotation of a crystal will produce a corresponding rotation in the EBSD pattern. The simplified geometrical model described does not consider the intensity associated with the EBSD patterns which also bear the information about the crystal structure. In particular, the kinematical theory of electron diffraction^11^ does not account for multiple scattering of electrons inside the crystal. Therefore for a quantitative calculation of the EBSD patterns, one has to consider the strong effects of multiple scattering and absorption, and hence dynamical theory of electron diffraction^11, 30^ becomes mandatory. Detailed reviews on various models for EBSD pattern simulations, limitations of kinematical theory and the physics behind dynamical theory calculations can be found elsewhere^11, 30–32^.

For the sake of simplicity, all the commercial EBSD systems determine crystal orientations with respect to the centrosymmetric Laue groups of a phase, although EBSD patterns are sensitive to the point-group symmetry of a crystal. Space group determination is also possible in certain cases which can be time consuming^33^. Advancement in computing power has made it possible to simulate the dynamical electron diffraction signal and compare it with the experimental patterns for the analysis of crystal orientation and their relationship with phase transformations and chirality determination^34–36^. In our present work, we have used the ESPRIT DynamicS (Bruker Nano) software which implements the Bloch wave approach for calculating the simulated EBSD patterns^30^. Typically 1000 or more reciprocal lattice vectors *hkl* (diffraction orders) are taken into account and they are selected according to the reciprocal lattice vector length d^*^_hkl_ = 1/d_hkl_ (typically d^*^_hkl_ < 1/0.05…1/0.035 nm^−1^) and the relative strength with respect to the largest structure factor amplitude |F| _max_ (typically < 10%). For interpreting the EBSD patterns and for plotting the orientation maps, the crystal orientations were parameterised using the ZXZ-type Euler angles (φ_1_, ϕ, φ_2_) in the Bunge convention^37^. For quantifying the agreement between two EBSD patterns, we have used the normalised cross-correlation coefficient, r^38, 39^.

EBSD patterns exhibit different distributions of intensity; an asymmetry in the intensity profile across a Kikuchi bands can be due to either the excess-deficiency effect^39^ or due to the breakdown of Friedel’s rule for the intensity at the symmetrically located Bragg angle locations, leading to ${I}_{hkl}\ne {I}_{\bar{h}\bar{k}\bar{l}}$ ^32^. It is this intensity asymmetry, due to the point-group sensitivity of EBSD, which we are going to exploit to image APDs. In contrast, the excess-deficiency effect is a result of the geometry of the measurement which affects the differential cross section for inelastic scattering^40^. The influence of the excess-deficiency asymmetry depends on the relative orientation of the Kikuchi bands with respect to the incident beam direction and can be minimized by careful selection of the sample orientation^41^. Please note the reliable discrimination of the Kikuchi bands intensity asymmetry is only possible when the intensity shift due to the breakdown of Friedel’s rule is considerably larger than the excess-deficiency effect.

## Results and Discussion

In non-centrosymmetric zincblende structures such as GaP, there is an asymmetric stacking sequence of Ga atoms and P atoms along $\langle 111\rangle$ and $\langle \bar{1}\bar{1}\bar{1}\rangle$ (see Fig. 1a)^33, 39^. Hence, Kikuchi bands formed from non-centrosymmetric lattice planes like {111} and $\{\bar{1}\bar{1}\bar{1}\}$ show an asymmetry in the intensity profile (i.e. the intensity maximum is marginally shifted out of the centre of the Kikuchi band) which allows the observation of the inversion symmetry^42, 43^. The effect of an asymmetry on all the polar lattice planes is demonstrated in Fig. 3. Figure 3a and b show the experimental EBSD pattern recorded from areas marked with a red dot and a purple dot (see inset of Fig. 1c) respectively. The corresponding simulated patterns are shown in Fig. 3d and e. We have used the automated best fit EBSD pattern matching approach^39^ based on the normalised cross-correlation coefficient r^38^ to compare the experimental EBSD patterns and the simulated EBSD patterns. On a casual assessment, both the experimental as well as the simulated EBSD patterns looks very similar. However, on careful inspection one can see the differences in the intensity associated with the Kikuchi bands, especially along the {111} bands where the higher intensity is towards either the top or bottom of the respective Kikuchi band edge which indicates the {111} and $\,\{\bar{1}\bar{1}\bar{1}\}$. This can be seen clearly in the normalised intensity difference (I_a_−I_b_)/(I_a_ + I_b_) (Fig. 3c) between the two experimental EBSD patterns (Fig. 3a and b). The correct orientation with respect to the point-group symmetry ($\bar{4}3\,$ *m*) is determined by best-fitting the experimental and the respective simulated patterns which provide the higher cross-correlation coefficient r. The Euler angle φ_2_ provides the last rotation around Z-axis which is equivalent to a rotation around [001] or the *c*-axis (which is also the epitaxial growth direction of our sample). The experimental EBSD pattern shown in Fig. 3a is compared with the simulated pattern shown in Fig. 3d and results in r of 0.627 and the Euler angle φ_2_ of 180.9°. But, when the Euler angle φ_2_ for the simulated EBSD pattern is changed to 270.9° (i.e. 180.9° + 90 °) the value of r is decreased to 0.599, representing a small but significant discrepancy from the experimental EBSD patterns. The same approach is repeated for the experimental pattern shown in Fig. 3b and the respective simulated EBSD pattern shown in Fig. 3e. In this case, when the Euler angle φ_2_ is of 180.9° the value of r is 0.601 (decreased when compared to Fig. 3a and d), but when the Euler angle φ_2_ is of 270.9° the value of r is increased to 0.627 (increased when compared to Fig. 3a and d). Therefore, for the experimental pattern shown in Fig. 3a, the correct experimental Euler angle φ_2_ is 180.9° whereas for the pattern showed in Fig. 3b, the correct experimental Euler angle φ_2_ is 270.9°.

The dissimilarity between the Kikuchi patterns with two different Euler angles can be best seen in Fig. 3c,f which show the normalised intensity difference (by using the same scaling) between the two experimental (Fig. 3a,b) and the corresponding simulated patterns (Fig. 3d,e). The sufficient intensity difference between polar planes such as {111} and $\{\bar{1}\bar{1}\bar{1}\}$ clearly indicates the 90° sub-lattice rotation confirming the presence of APDs in the GaP thin film. As all crystal directions normal to an even fold rotation axis are non-polar^41^, all the Kikuchi bands of {hk0}, for e.g. {110} disappear in the normalised difference intensity image (see Fig. 3c,f). In our case the asymmetric intensities are not due to excess-deficiency effects, nonetheless care has to be taken while acquiring the EBSD patterns as the strength of the excess-deficiency effects depends on the relative orientation of the Kikuchi bands with respect to the incident beam direction. Bands running normal to the incident beam direction (horizontal bands) show more of the excess-deficiency effects. Hence the Kikuchi bands of {111} and $\{\bar{1}\bar{1}\bar{1}\}$ are acquired diagonally in Fig. 3 when compared to the indexed EBSD pattern shown in Fig. 2b.

### Imaging BSE with an EBSD detector

Recently it was shown that it is possible to use the EBSD detector as an imaging device^44–46^ similar to a diode detector used in ECCI ^28, 29, 47^. Basically, each pixel of the CCD camera operates as an individual backscattered electron detector and the intensity of electrons at a specific pixel is recorded at each point during a step by step scanning of the sample, helping to derive the microstructural information^46, 48, 49^. We have utilised this feature and have defined regions which mainly covers the EBSD patterns formed by {111} and $\{\bar{1}\bar{1}\bar{1}\}$ (e.g. see Fig. 3a,b) as our regions of interest (ROI). This enabled us to map the asymmetrical intensity variations thereby revealing the APDs by calculating the intensity asymmetry (I_a_−I_b_)/(I_a_ + I_b_) between two ROIs, corresponding to reflections related by inversion. Figure 4a shows the Kikuchi bands ROI asymmetry image derived from the post processing of the recorded EBSD patterns^50^. Bright and dark regions correspond to opposite asymmetry values with an additional minor experimental offset caused by excess-deficiency effects and other intensity variations underlying the selected ROIs; and so allowing the respective APDs. The corresponding EBSD pattern quality image or in other words the total intensity image (Fig. 4b) shows the sample morphology, similar to the FSE image. Thus by choosing only a particular band as ROIs, quantitative microstructural information on the APDs can be obtained in a relatively simple way. This can be compared to the inverse pole figure (IPF) orientation maps as shown in Fig. 4c obtained from the same area using the automated pattern matching approach as discussed previously.

### Inverse pole figure (IPF) orientation maps

**Figure 5:**
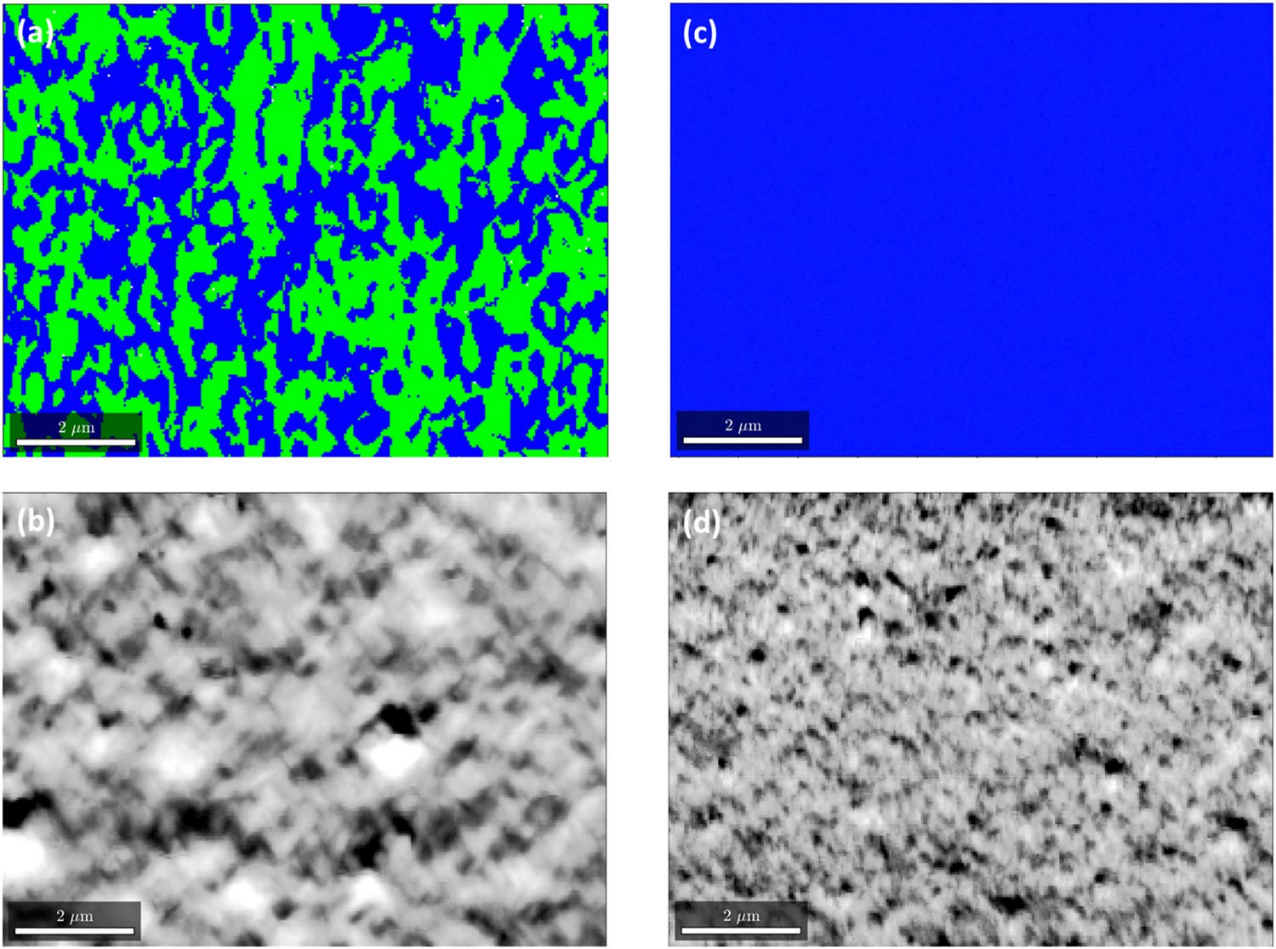
EBSD Inverse Pole Figure (IPF) maps of GaP thin films. (**a**) Grown on a different (001) Si substrate with similar growth conditions as the sample shown in Fig. 4.(b) corresponding BSE image derived from the EBSD patterns (**c**) IPF-map of GaP grown on a 4° misoriented Si substrate, not showing any APDs and (**d**) its corresponding BSE image derived from the EBSD patterns. The colour keys for the IPF-maps in (**a**) and (**c**) are the same as shown in Fig. 4c.

The IPF specifies the crystallographic description of a specific sample direction, i.e. it displays which lattice direction $\langle uvw\rangle$ is parallel to the sample direction the IPF is assigned to. To reveal the APDs using different colours in the IPF colour key, we have chosen the unconventional sample direction [1,1,1] as the reference (usually one selects X = [1,0,0], Y = [0,1,0] or Z = [0,0,1] as the reference directions). The maps show that the more general reference direction [1,1,1] is coloured by green regions, i.e. parallel to $\langle 1\bar{1}1\rangle$ or rotated by 90° around [001] so that $\langle 111\rangle$ is parallel to the [1,1,1] sample direction, the blue regions. The step pattern (arising due to the Si substrate steps) with a step width of ≈ 100 nm, similar to the dimensions of the atomic steps on the Si surface, can also be clearly seen along the $\langle 110\rangle$. This confirms the formation of APDs due to the steps along the (110) as explained previously (see Fig. 1a). The percentage of APDs can be estimated by calculating the areas of the green regions from a scanned area of ≈ 75 µm^2^, which accounts for ≈ 50%. The density of the APBs is estimated to be ≈ 2.6 µm^−1^. The shapes of the APDs seem to be non-uniform and appear to be narrow along $\,\langle 110\rangle$. Cross-section EBSD or 3-D EBSD could be useful for characterising the shape of the APDs for thicker samples. In order to check the reliability of our analysis, IPF maps were plotted for another GaP sample with similar growth conditions, however on a Si substrate from a different manufacturer (see Fig. 5a). The percentage of APDs is similar to the previous sample; with an APD content of ≈ 50% and an APB density of ≈ 2.7 µm^−1^. Figure 5b, shows the corresponding EBSD pattern quality image revealing the sample morphology. We have also performed experiments on a GaP sample grown on a 4° misoriented Si substrate which did not reveal any APDs (see Fig. 5c). This is expected because of the thermodynamically favored formation of bi-atomic steps on the Si substrates with a large (>4°) off-cut^51, 52^ preventing the formation of APDs in the GaP layers. The sample morphology derived from the EBSD pattern quality map is displayed in Fig. 5d. The APB densities estimated from our present work are similar to numbers derived from Barrett *et al*. on GaAs epitaxially grown on (001) Si^53^.

### Cross-correlation based high angular resolution EBSD

**Figure 6:**
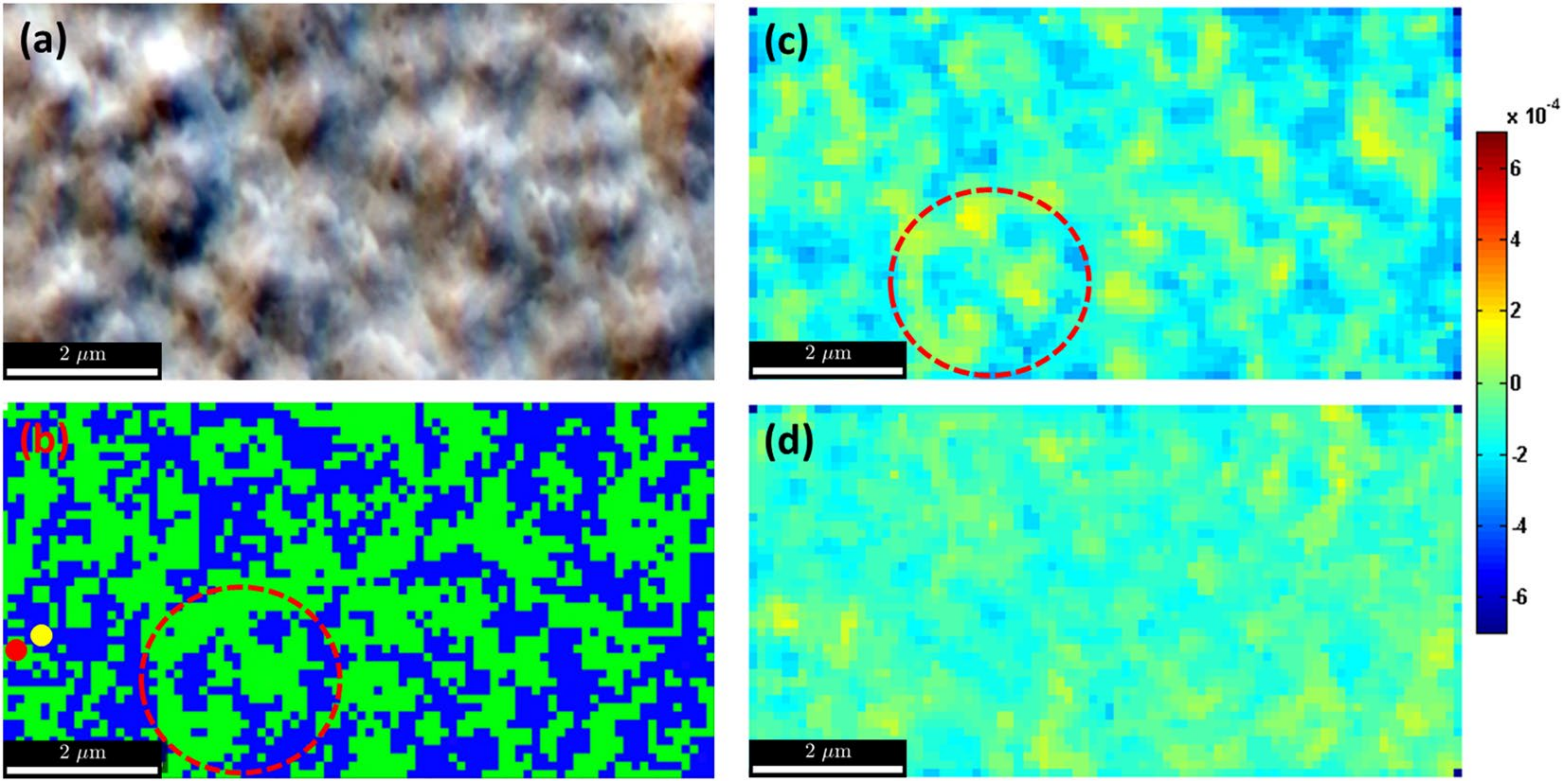
Comparison of IPF maps with cross correlation based MAE maps. (**a**) Forescatter image, (**b**) IPF map, **(c**) MAE (in radians) map plotted with green area in the IPF as a reference pattern, see red dot and (**d**)) MAE map with blue area as the reference pattern, see yellow dot.

Since the 90° lattice rotation around [001] makes it possible to image the APDs due to the asymmetric intensity in the Kikuchi bands, consequently it should also be possible to image them using cross-correlation based high angular resolution (HR)-EBSD^54, 55^. In HR-EBSD all the experimental patterns within a map are compared to a user selected reference experimental EBSD pattern using cross-correlation of the pattern intensities and the position within a number of ROI (35 ROIs in our present case) with a band pass filter applied in the Fourier domain to remove high frequency noise and low frequency background intensity variations. Cross-correlation algorithms find the translation between two matched regions within the patterns being compared and extract a translation (shift) along both ×_1_ and ×_2_ directions using the cross-correlation peak position. In addition, the correlation peak height is used as a measure of how good the best pattern matching is^56^. Maps of the variation of elastic strain (*ε*_*ij*_) and lattice rotation (*ω*_*ij*_) relative to that at the reference point (selected reference experimental EBSD pattern) can be generated using HR-EBSD with a very high precision of better than 10^-4^ rads (for rotation) and about 10^-4^ (for strain), respectively. More information about HR- EBSD cross-correlation analysis and its applications for strain and misorientations analysis are given in refs 54–57. In order to image the APDs, we have plotted the Mean Angular Error (MAE) map which is a quantitative measurement of unrealistic rotation measurements. Please note the 90° rotation around [001] between the sides of the boundaries is not the actual crystal misorientations. The MAE maps, where the mean of the errors for each ROI, between the as measured shift of a particular ROI and the shift expected from the finally calculated rotation tensor for that same ROI, can be used to image APDs. Figure 6a shows the FSE image of a GaP film (same as shown in Fig. 4) taken prior to acquiring HR-EBSD maps and Fig. 6b shows the corresponding IPF map plotted using the same procedure used to plot Fig. 4c. The cross correlation analysis of the HR Kikuchi patterns were conducted off-line and it is possible to choose the reference pattern from the area of interest of our choice. We have taken a reference pattern from the green region (APD region) marked with a red dot and plotted the MAE map; this can be seen in Fig. 6c. A dotted red circle is marked in both Fig. 6b and c to highlight the same area. We have also plotted the MAE map by choosing a reference pattern from the blue region (non-APD region) marked with a yellow dot. One can notice a broader distribution of MAE when the reference pattern is taken from the APD regions.

## Conclusion

In summary, the asymmetrical intensity associated with the EBSD patterns acquired using backscattered electrons in a scanning electron microscope is used to image APDs in GaP thin films grown on Si substrates. We have used three approaches to quantify and image APDs, namely by (i) comparing the experimental pattern with the dynamically simulated pattern, (ii) orientation mapping (IPF maps) using the correct non-centrosymmetric point group by involving an automatic pattern matching approach and (iii) plotting mean angular error maps using cross-correlation based HR-EBSD. We have also tested our automatic pattern matching approach on GaP samples grown on different Si substrates with and without APDs to show the reliability of our nanoscale, rapid and non-destructive approach on imaging APDs. The proposed analysis may well be generally applied for a wider range of other materials possessing non-centrosymmetric point groups.

## Methods

### Growth of GaP thin films

GaP films were grown on (001) Si substrates by metalorganic vapor phase epitaxy. The Si substrates were either exactly (001) oriented with a miscut < 0.1° or intentionally miscut by 4° towards $\langle 110\rangle$. Prior to the growth, the Si substrates were etched in HF: H_2_O (1:10) for 60 seconds to remove the native oxide followed by high temperature (i.e. 850 °C) *in-situ* annealing step in H_2_ environment. Tertiarybutylphosphine (TBP) and trimethylgallium (TMGa) were used as source materials. In order to ensure that the growth mode is as 2-D as possible, a two-step growth mode was used. First, a nominally 5 nm-thick GaP nucleation layer (NL) was grown at 475 °C. Due to reduced ad atom surface mobility at lower growth temperatures, the surface coverage of the ad atoms is improved causing the NL to cover the Si surface. However, due to inefficient decomposition of the source materials at lower growth temperatures, the growth rate of NL is extremely slow, approximately 10% of that at 600 °C. Therefore, the growth temperature was ramped up to 700 °C to grow a 70-nm-thick GaP layer on top of the NL. Note that the thickness of the GaP layer was selected to be 70 nm because it is smaller than the critical thickness for misfit dislocation formation. The sample surface shows the RMS roughness value from the AFM analysis is ≈ 1.5 nm for a 2.5 µm^2^ scanned area^49^.

### FE- SEM beam conditions and EBSD experimental settings

The SE image (Fig. 1b) and the BSE image (Fig. 1c) were recorded using an electron beam energy of 15 keV and a probe current of 5 nA with a sample tilt of 70 ° in a Merlin (Zeiss) FE-SEM. The same SEM is also used to obtain the experimental EBSD patterns shown in Figs 2b, 3a and 3b and EBSD maps displayed in Fig. 6. The BSE image shown in Figs 1c and 6a were taken combining the signals from three detectors, a multi-detector system (ARGUS ^TM^) which was positioned below the phosphor screen to collect the forward scattered electrons. The Kikuchi bands ROI asymmetry image (Fig. 4a) and the IPF maps (Figs 4c, 5a and 5c) were obtained using a Bruker e^-^Flash^HR+^ EBSD detector mounted on a LEO 1530VP (Zeiss) FE-SEM. The EBSD patterns were acquired using electron beam energy of 20 keV and a probe current of 5 nA. The detector to sample distance was 18 mm. The maps were obtained with a step size of 30 nm from EBSD patterns with a resolution of 160 × 115 pixels with 15 ms acquisition time. The mean angular error maps shown in Fig. 6c and d were also obtained using a Bruker e^-^Flash^HR+^ EBSD detector in a Merlin (Zeiss) FE-SEM with electron beam energy of 20 keV and a probe current of 5 nA with a detector to sample distance of 21 mm. The maps were acquired with a step size of 110 nm with a EBSD pattern resolution of 800 × 576 with 300 ms acquisition time.

### Cross-correlation coefficient (r)

For quantifying the agreement between two EBSD patterns, we have used the normalised cross-correlation coefficient r which can be defined by the below formula.

$${\rm{r}}=\frac{{\sum }_{i,j}\,[f(i,j)-\bar{f}]\,.[\omega \,(i,j)-\bar{\omega }]\,}{\sqrt{{\sum }_{i,j}\,{[f(i,j)-\bar{f}]}^{2}}.\sqrt{{\sum }_{i,j}\,{[\omega (i,j)-\bar{\omega }]}^{2}}}$$

where *f (i, j)* and *ω (i, j)* are the pixel intensity values of the corresponding ROI in the two EBSD patterns to be compared whereas $\bar{f}$ and $\bar{\omega }$ are the mean values in these ROIs. The absolute value of r is in the range between 0 and 1 and does not depend on scale changes in the intensity of both patterns. Values of r > 0.6 like those observed in this study indicate convincing fits between the experimental and simulated EBSD patterns^38^.

### Assessment of APBs density

We have estimated the APBs density based on number of boundaries (intersection between green to blue region from the IFP maps) that crosses along APDs. We have taken 5 line scans from random locations and counted the numbers of boundaries crossing the 10 µm in length and divided the number of boundaries by 10 µm, thus came up with an average APBs density of ≈ 2.6 µm^−1^ from an scanned area of ≈ 75 µm^2^.

### Data availability

The data that support the findings of this study can be found online under DOI: ﻿http://dx.doi.org/10.15129/2bb2bf6a-8ced-4c3f-812b-2b780f353b43 Alternatively, it is also available from the corresponding author on request.
